# Supplementary figures and images for: TNF-α Inhibitors Decrease Classical CD14hiCD16− Monocyte Subsets in Highly Active, Conventional Treatment Refractory Rheumatoid Arthritis and Ankylosing Spondylitis
Source: Int J Mol Sci. 2019 Jan 12;20(2):291. doi: 10.3390/ijms20020291 (PMC6358965; doi:10.3390/ijms20020291)

Supplementary Figure 1

A

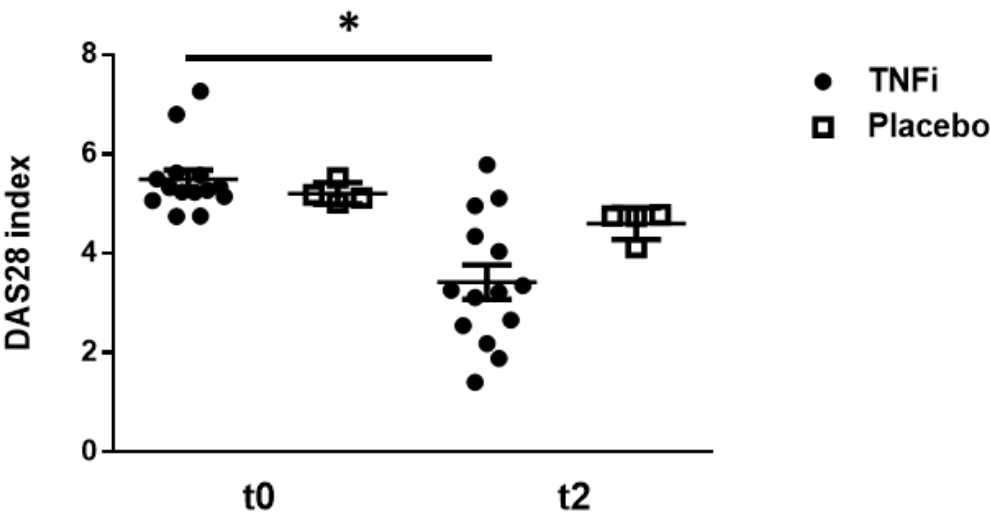

B

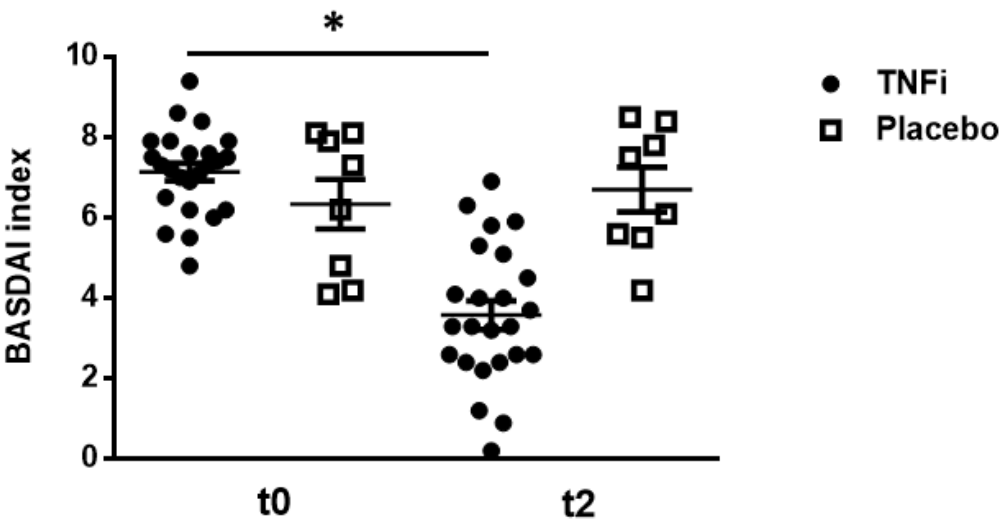

Supplement: Supplementary file 1 [file ijms-20-00291-s001.pdf]
